# Fast and Accurate Detection of COVID-19 Along With 14 Other Chest Pathologies Using a Multi-Level Classification: Algorithm Development and Validation Study

**DOI:** 10.2196/23693

**Published:** 2021-02-10

**Authors:** Saleh Albahli, Ghulam Nabi Ahmad Hassan Yar

**Affiliations:** 1 Department of Information Technology College of Computer Qassim University Buraydah Saudi Arabia; 2 Department of Computer Science Kent State University Kent, OH United States; 3 Depratment of Electrical and Computer Engineering Air University Islamabad Pakistan

**Keywords:** COVID-19, chest x-ray, convolutional neural network, data augmentation, biomedical imaging, automatic detection

## Abstract

**Background:**

COVID-19 has spread very rapidly, and it is important to build a system that can detect it in order to help an overwhelmed health care system. Many research studies on chest diseases rely on the strengths of deep learning techniques. Although some of these studies used state-of-the-art techniques and were able to deliver promising results, these techniques are not very useful if they can detect only one type of disease without detecting the others.

**Objective:**

The main objective of this study was to achieve a fast and more accurate diagnosis of COVID-19. This study proposes a diagnostic technique that classifies COVID-19 x-ray images from normal x-ray images and those specific to 14 other chest diseases.

**Methods:**

In this paper, we propose a novel, multilevel pipeline, based on deep learning models, to detect COVID-19 along with other chest diseases based on x-ray images. This pipeline reduces the burden of a single network to classify a large number of classes. The deep learning models used in this study were pretrained on the ImageNet dataset, and transfer learning was used for fast training. The lungs and heart were segmented from the whole x-ray images and passed onto the first classifier that checks whether the x-ray is normal, COVID-19 affected, or characteristic of another chest disease. If it is neither a COVID-19 x-ray image nor a normal one, then the second classifier comes into action and classifies the image as one of the other 14 diseases.

**Results:**

We show how our model uses state-of-the-art deep neural networks to achieve classification accuracy for COVID-19 along with 14 other chest diseases and normal cases based on x-ray images, which is competitive with currently used state-of-the-art models. Due to the lack of data in some classes such as COVID-19, we applied 10-fold cross-validation through the ResNet50 model. Our classification technique thus achieved an average training accuracy of 96.04% and test accuracy of 92.52% for the first level of classification (ie, 3 classes). For the second level of classification (ie, 14 classes), our technique achieved a maximum training accuracy of 88.52% and test accuracy of 66.634% by using ResNet50. We also found that when all the 16 classes were classified at once, the overall accuracy for COVID-19 detection decreased, which in the case of ResNet50 was 88.92% for training data and 71.905% for test data.

**Conclusions:**

Our proposed pipeline can detect COVID-19 with a higher accuracy along with detecting 14 other chest diseases based on x-ray images. This is achieved by dividing the classification task into multiple steps rather than classifying them collectively.

## Introduction

### Background

The COVID-19 pandemic has been causing significant health concerns since 2019. Symptoms of the disease include fever, cough, headache, and severe respiratory complications, which can subsequently lead to death. When this disease first started to spread in December 2019, numerous unknown facts were reported in Wuhan, China, where the first outbreak occurred [1]. By early January 2020, the government of China and the World Health Organization recognized SARS-CoV-2, the novel coronavirus known to cause COVID-19, as a pathogenic virus that belongs to the same family (Coronaviridae) as the virus known to cause severe acute respiratory syndrome (SARS). A SARS outbreak was previously reported in China in 2002-2003 [2].

Medical x-rays (short for x-radiation) are a form of visible light rays but with higher energy that penetrate the body to generate images of tissues and structures within the body, including bones, chest, and teeth. X-ray imaging is a very effective diagnostic tool and has been used for several decades by specialists to detect fractures, certain tumors, pneumonia, and dental problems [3]. In advanced cases, computed tomography (CT) can be used to produce a series of body images, which is later assembled into a 3D x-ray image that is processed by a computer. However, the traditional x-ray is a lot faster, easier, cheaper, and less harmful than a CT scan [4].

Research has shown that deep learning can be used to make predictions based on medical images by extracting characteristic features, including the shape and spatial rotation, from the images. Convolutional neural networks (CNNs) have played a very vital role in feature extraction and learning patterns that enable prediction. For example, a CNN is used to improve extraction high-speed video-endoscopy when the training data is very limited [5]. Advancements in image processing tools have brought about a radical change in the current techniques for the detection of pulmonary diseases. Researchers are employing traditional computer vision as well as deep learning algorithms to achieve satisfactory performance [3]. Several primary benefits are strongly correlated with the advancement of radiographic image classification tools. For example, in rural areas, owing to a shortage of doctors and places where doctors cannot be reached, such tools can prove useful. Once these tools become pervasive in the health care industry, radiologists, clinic practitioners, and even patients may utilize radiographic image classification tools to monitor and treat several diseases. As a result, this can reduce the burden on radiologists all over the world, by abolishing the requirement to examine every x-ray image for anomalies. Instead, the doctors will only need to focus on the patients whose x-ray images are flagged by this tool. The use of such tools can also eliminate the subjective opinion of doctors, increase the speed of early diagnosis of disease, and identify the minor details that may be overlooked by the human eye in some cases.

For this study, owing to computational restraints, we did not build a model from scratch, as such models require extremely high-end computers. Rather, we used CNN as a class of deep neural networks to propose a model to classify COVID-19 x-ray images from x-ray images of a wide range of chest diseases. Although the x-ray images of the other diseases are inadequate for proper training and to achieve state-of-the-art results, we generalized the data by considering data augmentation. This mainly rescales the x-ray images and flips them horizontally, in addition to a few other functionalities such as shift range, zooming, and rotation.

The strength of this study is that it classifies x-ray images at two different stages. The first stage involves enhancing the model to detect COVID-19–specific x-ray images at a faster speed than x-ray images of other chest diseases. This will result in a significant increase in the classification speed. Thus, considering a part of a dataset of chest x-ray (CXR) images for the analysis will result in low-quality output and unsatisfactory diagnoses. Accordingly, if the case is not classified as “normal” or “COVID-19” at this stage, then the classification is continued to the second stage, which involves classification for 14 other chest and related conditions (ie, atelectasis, cardiomegaly, effusion, infiltration, mass, nodule, pneumonia, pneumothorax, consolidation, edema, emphysema, fibrosis, pleural, and hernia). This also saves processing power if the x-ray image has been classified as “normal” or “COVID-19” in the first stage itself. To further enhance the accuracy of detection, we used UNet to complete lung and heart segmentation. Because we used a pretrained model, we were able to independently train 5 different models for each stage. Models with the best training and test accuracy were then selected for further analyses.

Based on our findings, we found that ResNet50 is the best model for classification in both scenarios: classifying 3 classes and 14 classes. Moreover, image segmentation helps in increasing the classification accuracy by up to 5%. We also trained a model for all 16 classes and found that classifying for a large number of classes significantly reduces the overall accuracy of the model.

The main contributions of this study are as follows:

1. Introduction of new classification pipeline for more accurate, automated classification in case of a large number of classes, primarily to increase the accuracy of a specific class.
2. Use of augmentation and semantic segmentation to increase accuracy of the model.
3. Comparison between different deep learning models on the basis of classification in cases of small and large number of classes.

In this paper, we first review previous studies that used deep neural networks for the detection of COVID-19 and other chest diseases. Then, we discuss the datasets used for our experiments as well as the study methodology, including data preprocessing, data segmentation, and the setup for classification of the models. Finally, we present the results and analyses on the basis of the models and dataset available.

### Previous Work

Recently, with the rapid development of artificial intelligence, an increasing number of researchers have begun to pay attention to intelligent, deep learning−based diagnostic techniques. Some of them have achieved significantly prominent results. In this section, we first review the current, state-of-the-art techniques concerning the application of artificial intelligence to chest diseases in general, and then, we discuss the literature related to COVID-19 detection using deep neural networks.

#### Detection of Chest Diseases Based on CXR Images by Using Deep Neural Networks

Sivasamy and Subashini [6] used a Keras framework to classify CXR images to predict lung diseases and reported an accuracy of 86.14%. The accuracy of the model improved as the number of epochs for training was increased. Wang et al [7] used pixel-wise annotated digital reconstructed radiograph data to train an unsupervised multiorgan segmentation model based on x-ray images. In this case, the gaps in nodules annotated directly on 2D x-ray images are quite challenging and time-consuming due to the projective nature of x-ray imaging. Rajpurkar et al [8] proposed a binary classifier for the detection of pneumonia from frontal-view CXR images that achieved an f1 score of 0.435. Salehinejad et al [9] used a Deep Convolutional Generative Adversarial Network (DCGAN) tailored model designed for training with x-ray images wherein a generator is trained to generate artificial CXR images. Their model obtained its best accuracy when trained on an augmented dataset with DCGAN-synthesized CXRs to balance the imbalanced real dataset (D3). Chandra and Verma [10] used 5 different models to identify pneumonia and reported 95.631% as the best accuracy. The model is limited to analyzing only nonrigid, deformable, registration-driven automatically lung regions and segmented region of interest–confined feature extraction. Previous studies using state-of-the-art techniques have achieved effective results with one or two cardiothoracic diseases, but these techniques could lead to misclassification.

A few techniques have targeted all 14 classes of chest diseases. Wang et al [11] presented the largest publicly available dataset of CXR images, which has provided a new dimension to the research community. They achieved promising results using a deep CNN and suggest that this dataset could be further extended by using more disease labels. Smit et al [12] proposed a deep learning−based technique to identify the 14 underlying chest diseases. They trained the model to input a single-view chest radiograph and output the probability of each of the 14 observations. Several models were trained to identify the one with the best accuracy. They used DenseNet121 for their research and found that it yielded the best accuracy, but it was limited to the CheXpert dataset and liable to overfitting. A pretrained DenseNet121 model and feature extraction techniques were used for accurate identification of 14 thoracic diseases in the study by Ho and Gwak [13].

#### Detection of COVID-19 Cases Based on CXR Images by Using Deep Neural Networks

There are several state-of-the-art studies on deep learning and machine learning models for COVID-19 diagnosis. A study by Apostolopoulos and Mpesiana [14] took advantage of CNNs for the automatic detection of COVID-19 by using CXR images. They adopted transfer learning to solve for the small image dataset challenge. Their COVID-19 dataset consisted of 224 sample medical images. Despite the size limitation, their results showed effective automatic detection of COVID-19−related diseases. Abbas et al [15] used the CNN-based DeTraC framework. They also used transfer learning to achieve the best performance. This model achieved 95.12% accuracy and 97.91% sensitivity. Chen et al [16] provided a prediction of patients with or without COVID-19 by using the UNet++ based segmentation model. Narin et al [17] classified CXR images using the ResNet50 model and obtained the highest classification performance with 98% accuracy, using a dataset comprising only 50 COVID-19 and 50 normal samples. Li et al [18] also used a ResNet50 model with a dataset comprising 468 COVID-19 samples, 1551 community-acquired pneumonia samples, and 1445 non-pneumonia samples; this model achieved 90% sensitivity. Using deep learning approaches to extract and transform features, Li et al proved their model’s efficacy in COVID-19 diagnosis [18]. Furthermore, Sethy and Behera [19] used deep learning to extract deep features from x-ray images and then used state vector machine to classify them into COVID-19–positive and COVID-19–negative classes; they achieved an accuracy of 95%. Hemdan et al [20] used transfer learning and fine-tuning on state-of-the-art networks like VGG and ResNetV2 to classify COVID-19–positive and COVID-19–negative x-ray images; they achieved an accuracy of 90%. Wang et al [21] proposed the M-inception model, a variant of the inception model. They detected only COVID-19 CT images from all available images and achieved an accuracy of 82%. Table 1 presents a comparison of previously studies models using radiographic imaging classification for COVID-19 cases, normal cases, and other chest diseases.

**Table 1:** Comparison of models detecting COVID-19 cases, normal cases, and other chest diseases based on medical images (data derived from [22]).

Reference	Medical image	Disease detected, n			Accuracy (%)	Methodology		Gaps in classification
		COVID-19	Normal	Other chest diseases				
Apostolopoulos and Mpesiana [14]	X-ray	224	504	700	93		Used transfer learning on VGG19. MobileNetV2, Inception, Xception, and InceptionResNetV2	Used only 3 classes: COVID-19, pneumonia, and other
Wang et al [23]	X-ray	53	8066	5526	92		Introduced COVID-Net—the first open-source COVID-19 detection system	Used only 3 classes: COVID-19, pneumonia, and normal
Narin et al [17]	X-ray	50	50	N/A^a^	98		Used 5 pretrained networks and applied 3 binary classifications for 4 classes of chest x-rays	Used only 3 classes: normal, COVID-19, viral and bacterial pneumonia
Brunese et al [22]	X-ray	250	3520	2753	97		Defined 2 models based on VGG16: one to classify affected x-ray images from healthy ones and the other to classify COVID-19 from affected x-ray images. Then, they localized the affected areas.	Although they used x-ray images of most diseases, they used only 3 classes: COVID-19, healthy, and disease
Song et al [24]	CT^b^	777	708	N/A	86		Proposed DRE-Net and compared its performance with VGG-16, DenseNet, and ResNet	Used only 3 classes: COVID-19, bacterial pneumonia, and healthy
Zheng et al [25]	CT	313	229	N/A	90		Proposed DeCoVNet for classification	Used only 2 classes: COVID-19–positive and COVID-19–negative
Xu et al [26]	X-ray	219	175	224	86		Proposed ResNet-18 based CNN^c^ network	Used only 3 classes: COVID-19, Influenza-A viral pneumonia, and normal
Ozturk et al [27]	X-ray	250	1000	500	92		Proposed DarkCovidNet	Used only 3 classes: COVID-19, pneumonia, and no findings
Ardakani et al [28]	CT	510	N/A	510	99		Used 10 CNN networks (ie, AlexNet and ResNet-101) for classification of 2 classes	Classified COVID-19 class from non–COVID-19 class
Li et al [18]	CT	1296	1325	1735	96		Proposed COV-Net for classifying 3 classes	Used only 3 classes: COVID-19, community-acquired pneumonia, and non-pneumonia
Abbas et al [15]	X-ray	105	80	11	95.12		Proposed DeTrac-ResNet18 CNN that uses Decompose, Transfer, and Compose architecture	Used only 3 classes: normal, COVID-19, and SARS
Chen et al [16]	CT	51	N/A	55	95.24		Used UNet++ along with Keras for segmentation and COVID-19 detection	Used only binary classification for COVID-19 detection

^a^N/A: not applicable.

^b^CT: computed tomography.

^c^CNN: convolutional neural network.

## Methods

### Dataset

The first step involved preprocessing of the data, which includes segmentation of the lungs and the heart from the whole image, as an x-ray image contains many unnecessary details. To perform this segmentation task, we trained the UNet model on segmented CXR data obtained by the Japanese Society of Radiological Technology, which were downloaded from their official website [29], and their corresponding masks, which were downloaded from the SCR database [30]. This dataset contains 247 images. For classification purposes, data for COVID-19 was collected from Cohen et al’s COVID Chest X-ray dataset [31]. This dataset contains x-ray images of many other diseases. Furthermore, x-ray images from the datasets were separated using the available metadata file. Data for the other 14 chest diseases were provided by the National Institute of Health (NIH) and can be downloaded from the NIH Chest X-ray Dataset of 14 Common Thorax Disease Categories [32]. Data available on the NIH Clinical Center website contains 112,120 images, belonging to 15 classes, which include 14 disease classes and 1 normal class—all of which were extracted through the available metadata file. The number of images per class is presented in Table 2.

**Table 2:** Number of images per class in the National Institute of Health Chest X-ray Dataset of 14 Common Thorax Disease Categories [32].

Model and class		Training set, n	Testing set, n
**Model 1**			
	COVID-19	455	22
	Normal	1995	405
	Other	4600	730
**Model 2**			
	Atelectasis	200	100
	Cardiomegaly	200	100
	Consolidation	200	100
	Edema	200	100
	Effusion	200	100
	Emphysema	200	100
	Fibrosis	200	100
	Hernia	150	100
	Infiltration	200	100
	Mass	200	100
	Nodule	200	100
	Pleural thickening	200	100
	Pneumonia	200	100
	Pneumothorax	200	100

The data were randomly split into training and testing sets, as there were very few data related to COVID-19. The idea was to keep the training set as large as possible given the small number of images present. Image augmentation compensated for the lack of data. This was not an issue for model 2 images. For model 1, however, the lack of data can cause a change in testing accuracy. To compensate for this issue, we also applied data augmentation while testing.

### Data Preprocessing

Every x-ray image has a different contrast and illumination as they are taken under different lighting conditions. Therefore, in the first step of preprocessing, histogram equalization was applied. CXR images also contain unnecessary details, such as the collarbone, shoulders, neck, and torso region. To remove these unnecessary details, lungs and heart segmentation were applied. For this purpose, the UNet segmentation model was trained on images from the Japanese Society of Radiological Technology with their corresponding masks. The architecture of the UNet model is shown in Table 3. The input image size fed to the network was 256×256×3. The contraction part acts as an encoder that extracts the context from the image using downsampling through the max-pooling layer. The expansive path acts as a decoder that precisely localizes the segmentation part using transpose convolution layers. It is an end-to-end, fully connected network and does not contain any dense layers. It also restores the image through upsampling.

**Table 3:** Architecture of UNet model.

Path, layer, and type			Kernel size	Filters		
	1	Input Layer	N/A^a^	N/A		
**Contraction Path**						
	2	Convolution	3×3	16		
	3	Dropout (0.1)	N/A	N/A		
	4	Convolution	3×3	16		
	5	MaxPooling	2×2	1		
	6	Convolution	3×3	32		
	7	Dropout (0.1)	N/A	N/A		
	8	Convolution	3×3	32		
	9	MaxPooling	2×2	1		
	10	Convolution	3×3	64		
	11	Dropout (0.2)	N/A	N/A		
	12	Convolution	3×3	64		
	13	MaxPooling	2×2	1		
	14	Convolution	3×3	128		
	15	Dropout (0.2)	N/A	N/A		
	16	Convolution	3×3	128		
	17	MaxPooling	2×2	1		
	18	Convolution	3×3	256		
	19	Dropout (0.3)	N/A	N/A		
	20	Convolution	3×3	256		
**Expansive Path**						
	21	Transposed convolution	2×2	128		
	22	Concatenate (21, 16)	N/A	N/A		
	23	Convolution	3×3	128		
	24	Dropout (0.2)	N/A	N/A		
	25	Convolution	3×3	128		
	26	Transposed convolution	2×2	64		
	27	Concatenate (26, 12)	N/A	N/A		
	28	Convolution	3×3	64		
	29	Dropout (0.2)	N/A	N/A		
	30	Convolution	3×3	64		
	31	Transposed convolution	2×2	32		
	32	Concatenate (31, 8)	N/A	N/A		
	33	Convolution	3×3	32		
	34	Dropout (0.1)	N/A	N/A		
	35	Convolution	3×3	32		
	36	Transposed convolution	2×2	16		
	37	Concatenate (36, 4)	N/A	N/A		
	38	Convolution	3×3	16		
	39	Dropout (0.1)	N/A	N/A		
	40	Convolution	3×3	16		
	41	Convolution (Sigmoid)	1×1	1		

^a^N/A: not applicable.

### Data Augmentation

Before feeding the data to the network, image augmentation was applied to tackle the problem of fewer data, that is, in the case of COVID-19. For applying augmentation, the rotation range was set to 90°, the horizontal flip was set to true, and the vertical flip was also set to true. For each iteration, the image data generator used a different transformation of the original images. In the case of COVID-19, we had 445 input images and 20 iterations; therefore, the data generator used 8900 images for training in this case.

### Classification Models

**Figure 1:**
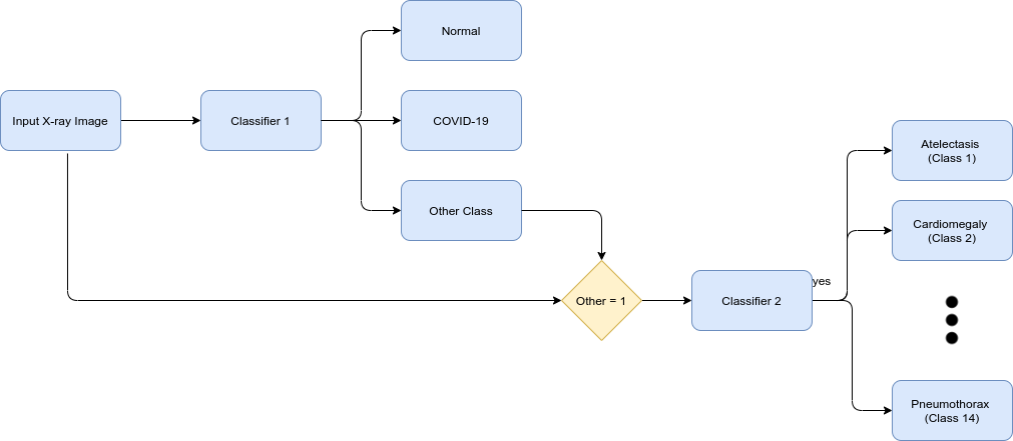
Proposed pipeline of classification.

The main objective of this study was to classify COVID-19 x-ray images from normal x-ray images and those of 14 other chest diseases. When a single model is trained for classifying 16 different classes, its accuracy tends to decrease, and in the case of COVID-19 detection, that is not acceptable. To solve this problem, a new pipeline was formed, which is illustrated in Figure 1. Two models were trained. The first model was trained to classify 3 classes: COVID-19, normal, and some other disease. The second model was trained to classify the 14 other chest and related diseases. Both models were trained separately. To automate the process, if the first model classified the x-ray as “some other disease,” then the second model was called to further classify the disease as one of 14 other chest diseases, using a simple “IF” condition. This architecture makes the classification process easy, as there are fewer features that need to be classified at the first stage.

Classifier 1 only needs to learn how to distinguish COVID-19 and normal x-ray images from those of all the other 14 chest diseases. The rule is simple: the fewer the classes, the fewer features there are to learn and distinguish, and the greater the accuracy. This is critical because the classification of COVID-19 is much more important than that of other diseases during the ongoing pandemic. Finally, the burden of classifying the other 14 x-ray diseases falls on classifier 2, which now has 14 classes to classify instead of 16. Furthermore, the 2 most important classes have already been classified by classifier 1. Moreover, to support the statement that accuracy indeed decreases when classifying into 16 classes, a third model was trained for classification into all 16 classes.

For classification purposes, the following 5 models were trained for both classifications:

NasNetLarge, Xception, InceptionV3, InceptionResNetV2, and ResNet50.

NasNetLarge was proposed by “Google Brain” in 2018 [33]. It has two types of architectures—CIFAR10 and ImageNet. CIFAR10 architecture has N number of normal cells and one reduction cell repeating after each other; in the end, it has the SoftMax function. ImageNet has two strides of convolutional layers with a 3×3 kernel size at the start, followed by two reduction cells; thereafter, it has the same architecture as CIFAR10.

Xception was proposed by Google in 2017 [34]. It consists of one entry flow, eight middle flow, and one exit flow. Entry flow consists of convolutional and max-pooling layers with ReLU as the activation function. The middle flow consists of only convolutional layers with the ReLU activation function. Exit flow consists of convolutional, max pooling, and global average pooling layers with the ReLU activation function; in the end, it has fully connected layers for classification.

InceptionV3 was proposed by Google in 2015 [35]. The basic architecture of the model is the same, as it consists of convolutional and pooling layers; in addition, it has three inception architectures as proposed previously [35]. Finally, at the end, it has the logistic and SoftMax function for classification into 1000 classes.

InceptionResNetV2 was proposed by Google in 2016 [36]. It has the proposed inception and reduction blocks at the start, and in the end, it has a pooling layer and dropout layer to prevent overfitting. It classifies using the SoftMax function.

ResNet50 was proposed by Microsoft in 2015 [37]. It takes residual learning as a building block and consists of convolutional layers with an average pooling layer at the end.

Models were taken from Keras library in Python, which were initialized with ImageNet weights. These models can classify 1000 classes, but we only needed to classify 3, 14, and 16 classes for classifier 1, classifier 2, and classifier 3, respectively. Therefore, these models were fine-tuned, and additional layers were added. Table 4 shows the fine-tuning layers added at the end of each pretrained model. The input image size given to the models was 331×331×3.

**Table 4:** Fine-tuning layers for classifier 1, classifier 2, and classifier 3.

Type	Classifier 1		Classifier 2		Classifier 3	
	Output	Kernel	Output	Kernel	Output	Kernel
Average pooling	2048	2×2	2048	2×2	2048	2×2
Flatten	8192	N/A^a^	8192	N/A	8192	N/A
Dense	1024	N/A	1024	N/A	1024	N/A
Dropout (0.5)	1024	N/A	1024	N/A	1024	N/A
Dense	1024	N/A	1024	N/A	1024	N/A
Dropout (0.5)	1024	N/A	1024	N/A	1024	N/A
Dense	3	N/A	1024	N/A	16	N/A

^a^N/A: not applicable.

All the models explained in the Methods section were trained and tested on Google Colab with 12 GB of RAM and GPU (graphics processing unit) assigned by Google Colab.

## Results

Initially, the UNet model was trained for segmentation of lungs and heart. After Training UNet, the model had a training loss of 16.75%, training accuracy of 87.13%, validation loss of 12.27%, and validation accuracy of 89.64%.

**Figure 2:**
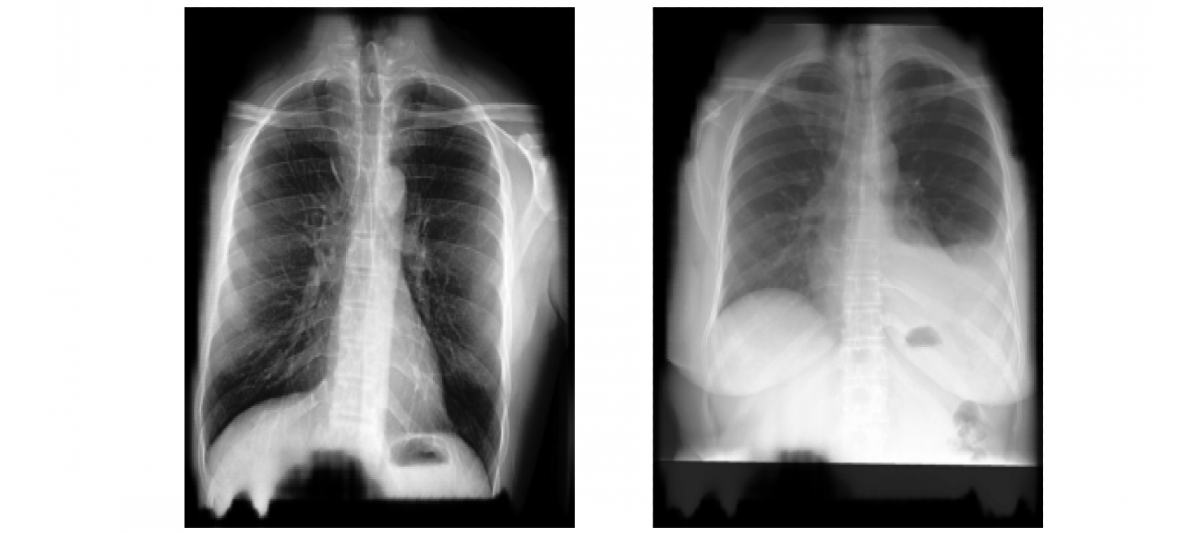
Sample segmented chest x-ray images.

Figure 2 shows some sample segmented CXR images. With image segmentation, we achieved up to 5% increase in the accuracy of our models.

After image segmentation was completed and new training data were obtained, each training model was trained for 20 epochs with a batch size of 8. The accuracy obtained from training is shown in Table 5. The table shows that the maximum accuracy for classifying the 3 classes, including COVID-19 was achieved by using ResNet50 followed by NasNetLarge. These two models yielded accuracy that competes with that of the available state-of-the-art models for COVID-19 prediction.

Classifier 2 did not show promising results in classifying the 14 other diseases. The main reasons for this were the large number of classes and the continued overfitting of the model. The maximum test accuracy achieved was 65.63% with ResNet50 followed by 61.47% with NasNetLarge.

As described, our proposed model pipeline helps to increase the accuracy of COVID-19 diagnosis when classifying 16 classes. Table 5 shows that when the 16 classes were combined and classified, the detection accuracy decreases. In all the cases except in the case of NASNetLarge and ResNet50 models, the test accuracy decreased when classifying 16 classes. Moreover, in the case of the NASNetLarge model, the increase in accuracy is not very notable. The maximum test accuracy was achieved with ResNet50, with an average of 71.905% over 10-fold cross-validation.

**Table 5:** Average training, validation, and test accuracy achieved by different models through a 10-fold cross-validation.

Model and classifier		Training accuracy (%)		Training loss (%)		Validation accuracy (%)		Validation loss (%)		Test accuracy (%)		Test loss (%)	
**NasNetLarge**													
	1st		91.80		33.78		91.25		31.32		89.66		32.028
	2nd		84.67		52.45		61.22		127.83		61.47		127.99
	Combined		79.72		68.36		63.68		123.42		63.58		121.39
**Xception**													
	1st		88.12		29.27		87.33		36.27		86.58		35.91
	2nd		90.70		48.73		61.88		133.04		61.08		133.92
	Combined		29.88		208.89		22.28		397.08		47.75		301.39
**InceptionV3**													
	1st		65.87		69.25		63.43		57.38		63.19		5.233
	2nd		83.91		56.46		53.57		142.22		53.75		139.97
	Combined		65.52		110.33		38.31		176.24		38.83		174.97
**InceptionResNetV2**													
	1st		65.10		80.32		62.46		76.35		63.19		75.79
	2nd		83.37		81.30		54.08		197.66		53.75		197.07
	Combined		54.45		134.20		33.84		200.61		33.97		200.26
**ResNet50**													
	1st		96.32		9.84		94.16		23.09		92.52		20.32
	2nd		87.83		35.85		67.55		105.63		65.63		108.24
	Combined		88.92		26.16		73.14		87.05		71.91		88.95

**Figure 3:**
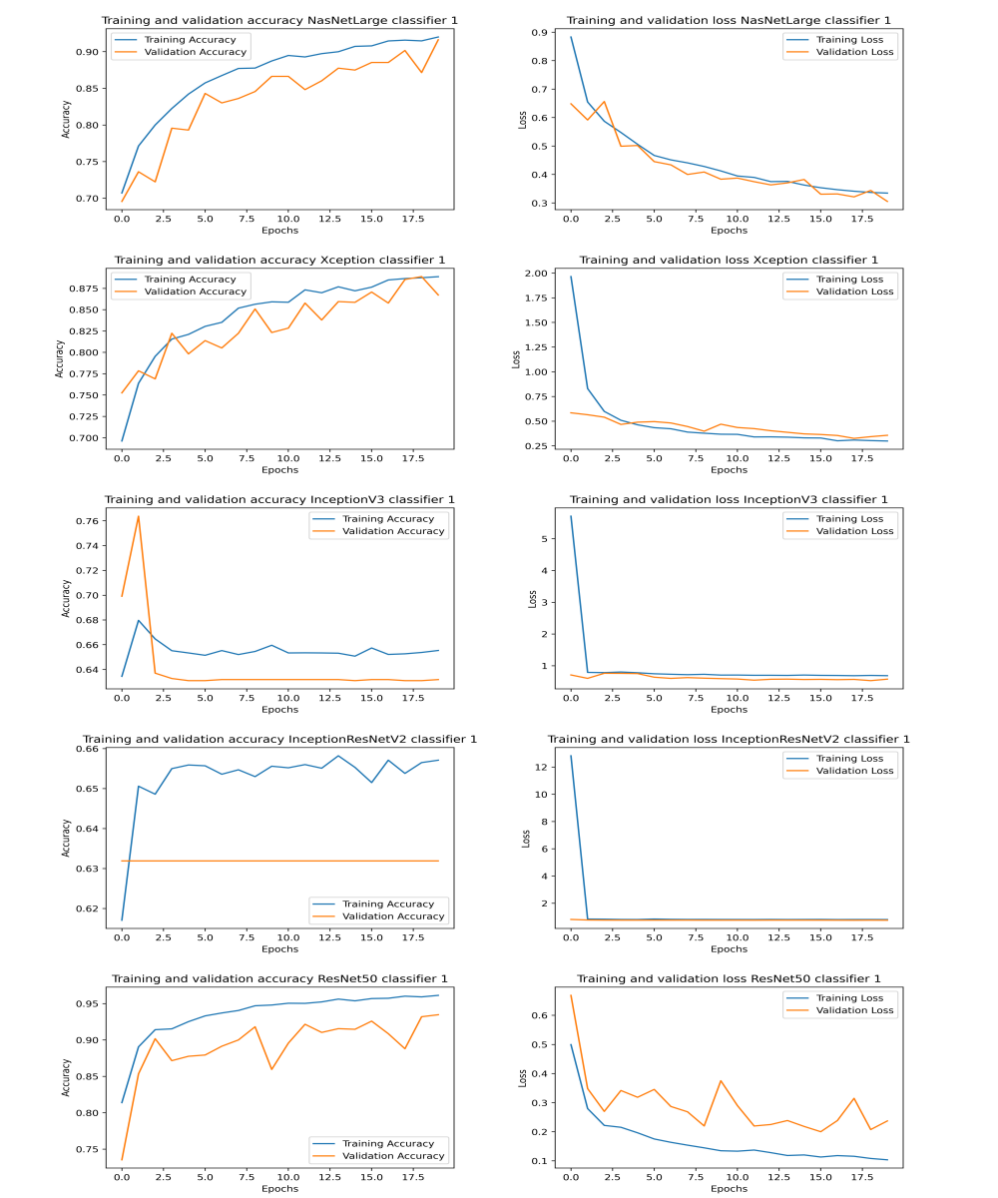
Graphs illustrating training and validation accuracy (left) and loss (right) over epochs for different models of classifier 1.

**Figure 4:**
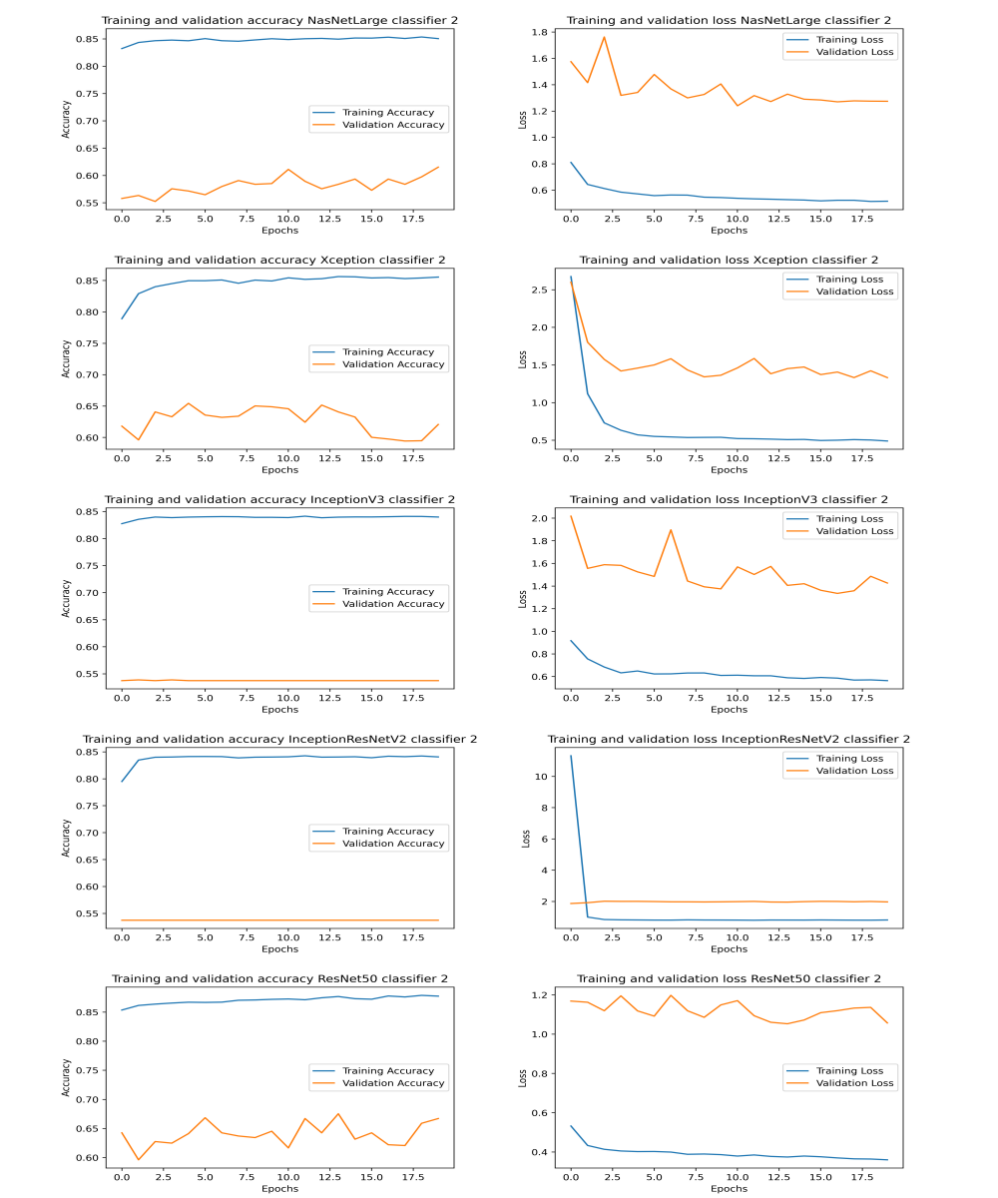
Graphs illustrating training and validation accuracy (left) and loss (right) over epochs for different models of classifier 2.

**Figure 5:**
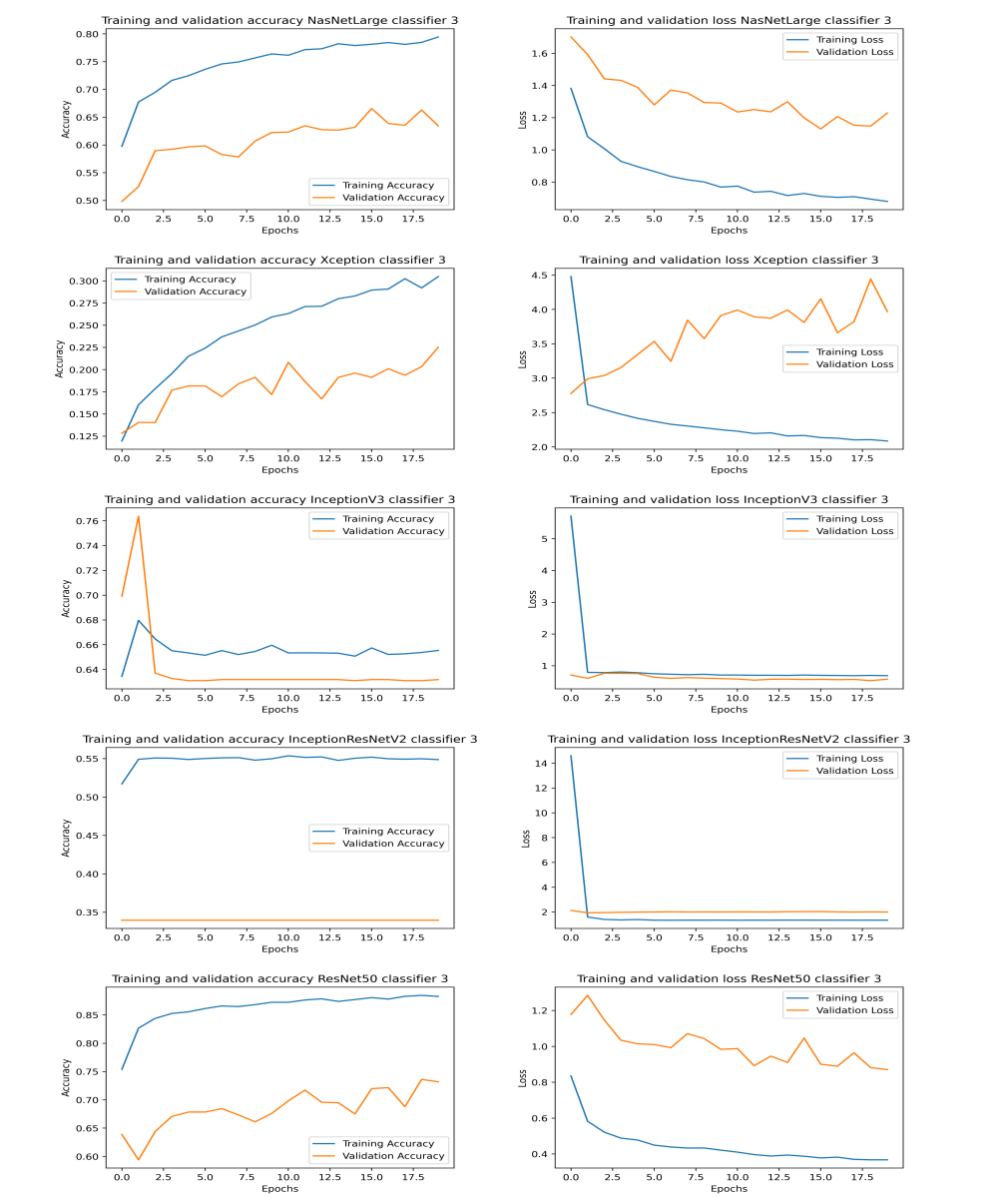
Graphs illustrating training and validation accuracy (left) and loss (right) over epochs for different models of classifier 3.

The results obtained by our proposed approaches compete with that of state-of-the-art methods (shown in Table 1). Graphs illustrating the training and validation accuracy and loss for classifiers 1, 2, and 3 are shown in Figures 3, 4, and 5, respectively. To further evaluate the results, the AUC (area under the curve), sensitivity, and specificity results for all the networks were studied (Table 6). We found that ResNet50 achieved the maximum AUC, sensitivity, and specificity scores compared to any other model.

**Table 6:** Average training, validation, and test accuracy achieved by different models through the 10-fold cross-validation.

Model and classifier		AUC^a^ (%)	Sensitivity (%)	Specificity (%)
**NasNetLarge**				
	1st	97.61	90.73	93.42
	2nd	82.15	75.33	81.1
	Combined	93.88	90.15	93.85
**Xception**				
	1st	95.9	88.64	91.78
	2nd	92.25	87.38	81.12
	Combined	83.19	76.09	80.64
**InceptionV3**				
	1st	89.28	83.2	85.51
	2nd	91.69	79.53	81.25
	Combined	89.85	83.29	93.7
**InceptionResNetV2**				
	1st	80.65	74.88	77.92
	2nd	85.41	79.21	81.25
	Combined	85.44	82.69	93.75
**ResNet50**				
	1st	98.73	93.14	95.22
	2nd	94.6	85.64	81.03
	Combined	96.9	93.4	93.72

^a^Area under the curve.

## Discussion

### Principal Findings

In this study, we classified normal cases, COVID-19 cases, and 14 other chest diseases based on CXR images. We proposed a novel, multiclass method for this purpose and used models that were pretrained on ImageNet dataset to save training time and resources. Our multilevel approach resulted in an increase in the classification accuracy. We found that ResNet50 was the best model for classification, yielding the highest accuracy.

### Future Suggestions

This study tried to cover most aspects of detection of chest diseases, but there is still work to be done. Most importantly, there is a need for more data for patients with COVID-19, which could help improve the accuracy of the model. At present, there is a significant difference in the number of images per class for the first level of classification.

This model can help in the first level of classification to determine whether the person has COVID-19 or some other chest disease, as x-rays are easier and less expensive than other forms of radiographic imaging and can help determine the severity of the disease. Although disease severity was not within the scope of this study, future work in detecting the severity of the disease can also be an important improvement in the already-existing model. In addition, techniques such as the Grad-Cam algorithm can be used to visualize the features in radiographic images affecting the algorithm and to determine disease severity. This algorithm will highlight which features help the algorithm with the classification and which features likely mislead the algorithm. This algorithm might also be the key to investigating the low accuracy of the level-2 classifier and can help improve its accuracy.

### Conclusions

Deep learning has played a major role in medical image analysis and feature extraction, which are applied to the detection of a wide range of chest diseases. CNN architectures are popular for their ability to learn mid- and high-level image representations and to make predictions. Detecting the presence, or absence, of COVID-19 in a patient is insufficient without addressing other chest diseases. However, a deep learning system that is trained to classify a large number of classes—16 in our case—has less accuracy. This work aimed to deal effectively with this new pipeline to help with a first-level differential diagnosis of COVID-19 from other chest diseases. Subsequently, we applied further enhancement to detect other chest diseases in order to tackle multi-class chest classification in the detection of anomalies on x-ray images. This approach yielded satisfactory results.

Thus, we showed how our proposed models use state-of-the-art deep neural networks to classify 16 cardiothoracic diseases by training the models based on x-ray images in the database. Image segmentation was applied to remove unnecessary details, and both classifiers were independently trained on segmented data. However, our model can classify not only COVID-19 but also 14 other chest diseases, as well as normal x-ray images, with satisfactory accuracy as compared with previous studies.

## Abbreviations

AUC: area under the curve
CNN: convoluted neural network
CT: computed tomography
CXR: chest x-ray
DCGAN: Deep Convolutional Generative Adversarial Network
NIH: National Institute of Health
SARS: severe acute respiratory syndrome
